# Towards the understanding of the cocoa transcriptome: Production and analysis of an exhaustive dataset of ESTs of *Theobroma cacao *L. generated from various tissues and under various conditions

**DOI:** 10.1186/1471-2164-9-512

**Published:** 2008-10-30

**Authors:** Xavier Argout, Olivier Fouet, Patrick Wincker, Karina Gramacho, Thierry Legavre, Xavier Sabau, Ange Marie Risterucci, Corinne Da Silva, Julio Cascardo, Mathilde Allegre, David Kuhn, Joseph Verica, Brigitte Courtois, Gaston Loor, Regis Babin, Olivier Sounigo, Michel Ducamp, Mark J Guiltinan, Manuel Ruiz, Laurence Alemanno, Regina Machado, Wilberth Phillips, Ray Schnell, Martin Gilmour, Eric Rosenquist, David Butler, Siela Maximova, Claire Lanaud

**Affiliations:** 1 Biological Systems Department – UMR DAP TA 40/03, CIRAD, Montpellier, France; 2 GENOSCOPE, 2 rue Gaston Crémieux, 91057 Evry, France; 3 CEPLAC, Km 22 Rod. Ilheus Itabuna, Cx. postal 07, Itabuna 45600-00, Bahia, Brazil; 4 Laboratório de Genômica e Expressão GênicaRodovia Ilhéus-Itabuna, UESC, Km 16, Ilhéus, Brazil; 5 USDA-ARS, 13601 Old Cutler Rd. Miami, Florida, USA; 6 Department of Horticulture, The Pennsylvania State University, 422 Life Sciences Building, University Park, PA, 16802, USA; 7 EET-Pichilingue, INIAP, Código Postal 24 Km 5 vía Quevedo El Empalme, Ecuador; 8 IRAD, Nkolbisson, BP 2067, Yaoundé, Cameroon; 9 UPR 31 TA 80/02, CIRAD, Montpellier, France; 10 UMR BGPI TA41/K, CIRAD- 34398 Montpellier France; 11 UMR BEPC TA 80/03, CIRAD, Montpellier, France; 12 MASTERFOODS, Almirante, Brazil; 13 CATIE, P.O.Box 7170, Turrialba, Costa Rica; 14 Mars Inc., Dundee Road, Slough, SL1 4JX, UK; 15 National Program Staff, USDA-ARS, Beltsville, Maryland 20705, USA; 16 Cocoa Research Unit, The University of the West Indies, St. Augustine, Trinidad and Tobago

## Abstract

**Background:**

*Theobroma cacao *L., is a tree originated from the tropical rainforest of South America. It is one of the major cash crops for many tropical countries. *T. cacao *is mainly produced on smallholdings, providing resources for 14 million farmers. Disease resistance and *T. cacao *quality improvement are two important challenges for all actors of cocoa and chocolate production. *T. cacao *is seriously affected by pests and fungal diseases, responsible for more than 40% yield losses and quality improvement, nutritional and organoleptic, is also important for consumers. An international collaboration was formed to develop an EST genomic resource database for cacao.

**Results:**

Fifty-six cDNA libraries were constructed from different organs, different genotypes and different environmental conditions. A total of 149,650 valid EST sequences were generated corresponding to 48,594 unigenes, 12,692 contigs and 35,902 singletons. A total of 29,849 unigenes shared significant homology with public sequences from other species.

Gene Ontology (GO) annotation was applied to distribute the ESTs among the main GO categories.

A specific information system (ESTtik) was constructed to process, store and manage this EST collection allowing the user to query a database.

To check the representativeness of our EST collection, we looked for the genes known to be involved in two different metabolic pathways extensively studied in other plant species and important for *T. cacao *qualities: the flavonoid and the terpene pathways. Most of the enzymes described in other crops for these two metabolic pathways were found in our EST collection.

A large collection of new genetic markers was provided by this ESTs collection.

**Conclusion:**

This EST collection displays a good representation of the *T. cacao *transcriptome, suitable for analysis of biochemical pathways based on oligonucleotide microarrays derived from these ESTs. It will provide numerous genetic markers that will allow the construction of a high density gene map of *T. cacao*. This EST collection represents a unique and important molecular resource for *T. cacao *study and improvement, facilitating the discovery of candidate genes for important *T. cacao *trait variation.

## Background

*Theobroma cacao *is a diploid species (2n = 2X = 20) with a small genome size of 380 Mbp [1,2]. It is a tree fruit originating from the tropical rainforest of South America. According to Cheesman (1944) [3], its center of origin is the lower eastern equatorial slopes of the Andes. *T. cacao *is now cultivated in all tropical lowlands of the world and its beans are used to produce chocolate and cocoa butter after a post harvest treatment including fermentation, drying and torrefaction steps. *T. cacao *is one of the major cash crops for several tropical countries. Its economic importance is high and presently cocoa is the third most important internationally traded raw material after sugar and coffee.

Cocoa is mainly produced on smallholdings. It is estimated that approximately 14 million people around the world rely on cacao plantations for income. *T. cacao *production is seriously affected by several fungal diseases and insect attacks. Oomycetes and especially *Phytophthora*, spp., (black pod) are responsible, worldwide, for 30% of losses. Several species are involved. *P. palmivora *is present in the entire cacao growing area, whereas *P. capsici *and *P. citrophthora *are prevalent in South America. *P. megakarya *is limited to some countries in West Africa, however it is by far the most aggressive species causing losses of production up to 50% Harvest losses due to *Phytophthora *species were estimated to be 450,000 tons [4].

Two basidiomycetes, *Moniliophthora roreri *(frosty pod) and *Moniliophthora perniciosa *(witches' broom) are also responsible for important harvest losses. In Brazil, *M. perniciosa *was responsible for a drastic yield loss with a fall in production from 405,000 tons in 1986 to less than 130,000 tons in 1998. *Moniliophthora roreri *causes a very destructive pod rot and has already had dramatic effects in some countries such as Ecuador [5] and Costa Rica [6]. *M. roreri *was confined to several countries of Central and northern South America, but is continuously spreading towards other Central American countries like Mexico or southward towards countries like Peru.

Several sources of disease resistance have been identified in different genetic backgrounds, and the search for a sustainable disease resistance, cumulating the different resistance genes is one of the major challenges of *T. cacao *genetic breeding programs [7].

Other traits of importance in *T. cacao *are quality traits. Food quality improvement, nutritional as well as organoleptic, is now a strong demand of consumers. Fundamental knowledge of the genetic basis of quality is an important challenge that can address this demand.

Flavor is among the main criteria of quality for chocolate manufacturers, but these characteristics are largely understudied by the cocoa research and breeding community due to their complexity and a dramatic lack of fundamental knowledge about these traits. Flavour components depend strongly on conditions of post-harvest processing [8]. After pod harvests, fresh seeds need to be fermented for 4 to 6 days, then dried and roasted to develope good cocoa aromas. Raw seeds, embedded in a pulp rich in sugar, undergo biochemical changes under the effect of various microorganisms present in the environment. The initial anaerobic, low pH and high sugar conditions of the pulp favour yeast activity, converting sugars in the pulp to alcohol and carbon dioxide. Bacteria then start oxidising the alcohol into lactic acid and then into acetic acid as conditions become more aerobic. These biochemical changes are accompanied by changes of amount and composition of several compounds having a major effect on cocoa flavor such as peptide aroma precursor formation, procyanidines or terpenes content.

However, it is now well recognized that the genetic origin is also a strong determinant of flavor, independent of the conditions of post-harvest processing [9].

Although some aromas are prominently defined by a single molecule, most aromas are composed of a bulk of volatile compounds responsible for aroma perception, and belonging to different classes of organic compounds. Interestingly, despite the vast number of chemical structures involved, the large majority of scent compounds are biosynthesized by a surprisingly small number of metabolic pathways. Parts of these metabolic pathways are ubiquitous, and have been developed by small but important modifications of ancestral genes and pathways [10]. In *T. cacao *more than 500 volatile compounds have been detected. However, only a small number are thought to play a key role in natural aroma variations.

Cocoa is classified into two classes: the «standard quality cocoa» corresponding to 95% of the total market, and the «fine flavor cocoa» produced by *T. cacao *trees originated from two main varieties: Criollo and Nacional, which bring a higher price in the market.

An important class of volatile compounds, the terpenes, plays an important role in the aromatic flavor of these varieties.

For example, a high level of linalool, a monoterpene, has been observed in Nacional varieties [11] from Ecuador, characterised by a floral taste, and could be at the origin of this specific flavor which represents an important economic «niche» for the country. However, the modern and hybrid Nacional varieties present a wide range of flavor variations due to introgressions of foreign and more vigorous varieties, leading to a dilution of this specific floral flavor, and recently a part of Ecuador cocoa production was declassified from fine flavor to "bulk cocoa" with a lower price. An increased knowledge of the metabolic pathways and expression of genes involved in terpene synthesis could help to improve the aromatic flavor of new "Nacional" varieties.

Independent to volatile compounds, some other biochemical compounds are known to interact with *T. cacao *organoleptic traits. This is the case with polyphenols. Catechin, epicatechin and procyanidines are the main polyphenols present in *T. cacao*. They have well known antioxidant biological activities and beneficial effects on the cardiovascular system [12-14]. Contributing to bitterness and astringency, polyphenols influence *T. cacao *organoleptic quality [15,12]. They influence aromatic profiles of *T. cacao *in restricting Maillard's reactions, which generates a majority of the aromatic compounds of *T. cacao*.

Genomic research provides new tools to study the genetic and molecular bases of important trait variations: EST sequencing projects carried out on other plant models have allowed the characterization of the transcriptome and facilitated the gene discovery of important trait variations [16]. In tree crops, except for poplar whose genome has been recently sequenced [17], genomic resources are generally limited, and few large EST collections have been produced. Recently, a *citrus *EST collection comprising 15,664 putative transcription units [18] has been produced, allowing the identification of clusters associated with fruit quality, production and salinity tolerance. A cotton study identified 51,107 unigenes from a global assembly of 185,000 cotton ESTs, [19] providing a framework for future investigation of cotton genomics. The same approach was used to characterize the grape transcriptome during berry development by the analysis and annotation of 25,746 unigenes from 146,075 ESTs [20].

In *T. cacao*, only small collections of ESTs have been produced so far and used to study gene expression related to stress or disease resistance and defense [21-24]

The objective of this study was to produce a large *T. cacao *EST collection from a wide range of organs, providing a good representation of *T. cacao *genes expressed during *T. cacao *development and suitable for further analysis of all kind of traits in *T. cacao*. Moreover, we emphasized the production of tools to further study *T. cacao *diseases, a major constraint for cocoa production, and quality features. Therefore, we also produced cDNA libraries relevant to disease resistance and quality traits. ESTs were produced from *T. cacao *tissues interacting with various pest and fungal diseases, from seeds at different stages of development and during the fermentation steps. This large EST collection will provide valuable tools to carry out functional genomic studies and discover genes essential to important agronomic and quality trait variation in *T. cacao*, aiming to accelerate *T. cacao *improvement. A multidisciplinary approach combining functional genomic and quantitative genetic approaches could lead to a better understanding of gene function involved in disease resistance mechanisms or quality trait variations. *T. cacao's *phylogenetic proximity to the model plant *Arabidopsis *will facilitate our understanding of most metabolic pathways. However, *T. cacao *is a tree, and expresses traits not found in *Arabidopsis*, thus we hypothesize that genes not found in *Arabidopsis *play important roles in cacao development.

## Results and Discussion

### Library construction

Fifty-six libraries were constructed from two main genotypes representing three contrasting genetic origins: ICS1, a hybrid between Criollo and Forastero from Lower Amazonia of Brazil, and Scavina 6, a Forastero from Upper Amazonia of Peru. A few other genotypes characterized by specific resistance or quality traits and belonging to various genetic origins were also used. The plant materials were provided from a various panel of different *T. cacao *L. organs (Table 1). Among them, 25 libraries corresponded to *T. cacao *tissues introduced to different biotic stresses: pods inoculated by *Phytophthora palmivora, Phytophthora megakarya, Moniliophthora perniciosa *and *Moniliophthora roreri*, leaves inoculated by *Phytophthora palmivora *and *Phytophthora megakarya*, stems inoculated by *Moniliophthora perniciosa *and *Ceratocystis fimbriata*, and stems attacked by *Sahlbergella singularis *(mirids). Among these libraries, 17 are suppressive subtractive hybridization (SSH) libraries. Finally, two libraries corresponded to *T. cacao *tissues introduced to drought stresses and 11 corresponded to seed development and fermentation stages.

**Table 1:** Summary of *T. cacao *libraries

**Genotype**	**Library**	**Library description**	**Good quality ESTs**	**Unigenes**
Jaca	CERATOJ_KZ0ACI	stem tissues inoculated by Ceratocystis fimbriata	1729	1270
Scavina6	CHERELS_KZ0AAC	cherels from 1 week to 1 month stage of development	4252	2836
				
Scavina6	COPHAS_KZ0AAL	pod tissue inoculated by Phytophthora palmivora	4905	2621
Scavina6	CORTEXS_KZ0AAT	cortex tissue, external part	3817	2227
Scavina6	CORTINS_KZ0AAV	cortex tissue internal part with lignified chanels	5096	3331
ICS1	COSSHPPI_KZ0AA	SSH library from tissues inoculated/non inoculated by Phytophthora palmivora	1721	955
				
Scavina6	COSSHPPS_KZ0AA	SSH library from tissues inoculated/non inoculated by Phytophthora palmivora	1702	1129
ICS1	COTYLEI_KZ0ABB	cotyledons from germinated seeds (1 to 3 weeks)	5153	2961
B97 C-C-2	CUSHIONC_KZ0ACAC	young cushions	2849	2120
Scavina6	DROUGHTLS_KZ0ACAF	leaves submited to drought stresses	2766	1290
Scavina6	DROUGHTRS_KZ0ACAE	roots submited to drought stresses	2685	1563
ICS1AF	EMBR1WI_KZ0ABA	epicotyle and hypocotyle from 1 week germinated seeds	3246	2473
				
ICS1AF	EPIC23I_KZ0AAS	epicotyle from 2–3 week germinated seeds	3005	2459
Scavina6	FLOWERS_KZ0AAD	flowers at different stages of development	3511	2434
Scavina6	FLPOLSSH_KZ0ABL_M	SSH library from ovaries submitted to compatible/incompatible pollinations	2398	431
ICS1AF	HYPO23I_KZ0AAP	hypocotyle from 2–3 week germinated seeds	5111	2955
Scavina6	LEAVES_KZ0ABE	young and adult leaves at different stages of development	4698	3069
				
GU255V	LEAVPAGU_KZ0ACQ	leaves inoculated by Phytophthora palmivora	3030	2139
PNG seedlings	LEPAPNGR_KZ0ACP	leaves inoculated by Phytophthora palmivora	1021	862
PNG seedlings	LESSHMEPNGa_KZ0ACAP	SSH library from leaves inoculated by Phytophthora megakarya from susceptible-resistant PNG seedlings	356	169
PNG seedlings	LESSHMEPNGb_KZ0ACV	SSH library from leaves inoculated by Phytophthora megakarya from resistant – susceptible PNG seedlings	1244	749
				
PNG seedlings	LESSHPNGRSb_KZ0ABP	SSH library from leaves inoculated by Phytophthora palmivora from resistant – susceptible PNG seedlings	701	438
UF676	MIRIDUFS_KZ0ACAD	young shoot tissues attacked by Sahlbergella singularis (mirids)	3011	1908
				
P7	MONILIOP_KZ0AB	pod tissues inoculated by Moniliophthora roreri	3074	2217
UF273	MONILIOU_KZ0ABV	pod tissues inoculated by Monilia roreri	3159	1871
IMC47	OVUL1_7M_KZ0ACAK	ovaries from 1 to 7 days after pollinations	1565	1218
ICS1	OVULEI_KZ0AAB	ovules collected 2 to 3 months after pollination	4942	3315
UPA134	PODMEUPA_KZ0ACAB	pod tissues inoculated by Phytophthora megakarya	3492	2093
Scavina6	PODSSHWB1Sb_KZ0ACD	SSH library from pod tissues inoculated-non inoculated by Moniliophthora perniciosa less than 60 days after inoculation	652	534
				
Scavina6	PODSSHWB2Sb_KZ0ACF	SSH library from pod tissues inoculated-non inoculated by Moniliophthora perniciosa between 60 to 120 days after inoculation	1399	912
Scavina6	PODWB1S_KZ0ACM	pod tissues inoculated by Moniliophthora perniciosa less than 60 days after inoculation	1704	1213
Scavina6	PODWB2S_KZ0ACN	pod tissues inoculated by Moniliophthora perniciosa between 60 to 120 days after inoculation	1718	1217
PNG seedlings	RESSHMEPNGb_KZ0AC	SSH library from leaves of resistant seedlings inoculated-non inoculated by Phytophthora megakarya	1287	931
Scavina6	ROOTS_KZ0ABF	roots	3567	2892
PNG seedlings	RPPSSHPNGa_KZ0ACAL	SSH library from leaves of resistant seedlings non inoculated- inoculated by Phytophthora palmivora	344	266
PNG seedlings	RPPSSHPNGb_KZ0ACR	SSH library from leaves of resistant seedlings inoculated-non inoculated by Phytophthora palmivora	1407	823
ICS1	SEED34I_KZ0AAH	seeds 3 to 3,5 months after pollinations	3942	2637
ICS1	SEED45I_KZ0AAE_F	seeds 4 to 5 months after pollinations	3296	1902
33–49	SEEDFERB_KZ0ACAG	Cotyledons from seeds fermented between 6 H and 4 days	1664	465
				
ICS1	SEEDMAI_KZ0AAG	seeds from mature pods 5,5 to 6 months after pollinations	3068	1844
				
BE240	SEEDNAB_KZ0ABH	seeds 2 to 5 months after pollinations	4988	3101
ICS1	SEFERMI_A_KZ0AAR	fermented seeds during 6 to 26 H	1798	844
ICS1	SEFERMI_B_KZ0AAM	fermented seeds during 32 to 40 H	3931	2110
Jaca	SSHCERATOJb_KZ0ACS	SSH library from stems inoculated-non inoculated by Ceratocystis fimbriata	339	327
Jaca	SSHCERATOJa_KZ0ACAM	SSH library from stems non inoculated-inoculated by Ceratocystis fimbriata	1364	918
UF676	SSHMIRUFa_KZ0ACAN	SSH library from young shoots non attacked-attacked by Sahlbergella singularis	320	296
UF676	SSHMIRUFb_KZ0ACT	SSH library from young shoots attacked-non attacked by Sahlbergella singularis	1393	1051
Scavina6	STEMS_KZ0AAA	complete disc of stems 1 cm diameter	4938	2880
Scavina6	STSSHWB1S_KZ0ABI_K	SSH library from (and reverse sens) shoot tissues inoculated/non inoculated by Moniliophthora perniciosa less than 18 days after inoculation	1594	370
				
Scavina6	STSSHWB2Sb_KZ0ACB	SSH library from shoot tissues inoculated-non inoculated by Moniliophthora perniciosa between 18 to 120 days after inoculation	1408	1056
				
33–49	TEGFERB_KZ0ACAH	testa from seeds fermented between 6 H and 4 days	1649	808
ICS1	TEGPULI_KZ0AAI_K	testa with pulp from mature seeds	5017	3254
Scavina6	TISCIVS_KZ0AAQ	embryogenic and non embryogenic callus in vitro culture	3434	2389
				
ICS1	TPFERMI_A_KZ0AAN	fermented testa during 6 to 40 H	4005	2164
P7	WILTP_KZ0ACL	young wilted cherels 7 to 10 days after pollination	1706	1247
Scavina6	WOODS_KZ0ACAA	bark and cambium part of wood	3478	2234

### EST sequencing and assembly

From the 56 libraries, 8565 clones were first sequenced on both strands using forward and reverse primers, to have an overview of the quality of the libraries, and then 163,868 clones were single-pass sequenced from 5' or from 3' end (Table 1). This represented a total number of 180,998 chromatograms that were used in this analysis. After low quality, vector and adapters trimming, 149,650 sequences longer than 100 bp remained as good quality sequences. The average sequence length was 472 bp and 62% were longer than 400 bp. These individual ESTs (available through EMBL-Bank [25]) were assembled using the TIGR Gene Indices clustering tools (TGICL) [26]. The assembly process produced 12,692 contigs and 35,902 singletons that represented a total of 25.6 Mb of transcripted sequences. The combined set of contigs and singletons resulted in 48,594 unigenes which might correspond to different putative transcripts or different parts of the same transcript found in the *Theobroma cacao *transcriptome. The average length of this *T. cacao *non redundant sequences dataset was 527 bp.

An assembly of ESTs has already been published for *Theobroma cacao *but has been limited to 1380 unigenes (4433 ESTs) from two leaf and bean cacao libraries [21], to the isolation of 1256 unigenes (2114 ESTs) from cacao leaves treated with inducers of defense response [23] and to 2926 non redundant sequences from libraries of cacao meristems inoculated by *Moniliophthora perniciosa *[24].

The results of this study are more comparable to a cotton EST project [19], involving 30 cDNA libraries. This analysis detected 51,107 unigenes in approximately 185,000 *Gossypium *ESTs.

**Figure 1:**
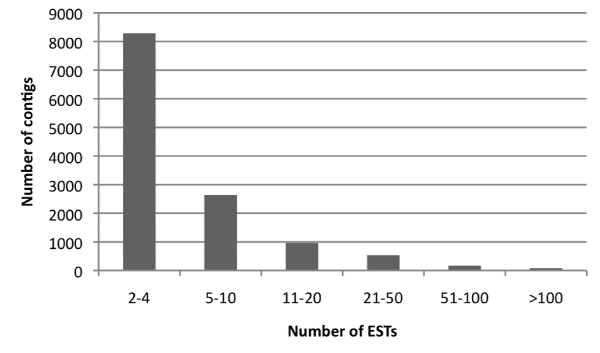
Distribution of *T. cacao *EST members in contigs after the assembly process.

Analysis of EST abundance in a contig can provide insights to gene expression levels, although this information must be taken with caution due to cloning and replication bias resulting form library construction and propagation steps. The number of ESTs in the *T. cacao *contigs ranged from 2 to 5102 (Figure 1) and 65.3% were composed of 4 or less ESTs. 98% of the contigs contained less than 50 ESTs.

**Figure 2:**
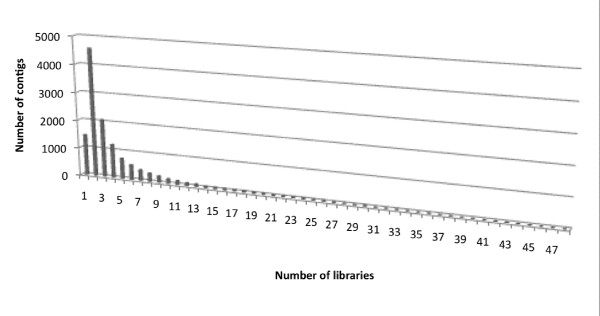
Number of contigs composed from sequence originated from one ore more libraries.

We evaluated the redundancy of transcripts in each library and among all libraries by studying the distribution of ESTs in contigs across multiple libraries. 11,226 had members from more than one library (Figure 2) and 1466 contigs were specific from one library. No contigs had members from all 56 libraries. Two contigs were found in 52 libraries: the contig CL1Contig269 was similar to the mitochondrial large subunit ribosomal RNA gene and the contig CL1Contig513 to the 18S ribosomal RNA gene. The contig CL18Contig2, CL2Contig3 and CL15Contig2, similar to an ATP Synthase beta subunit, a metallothionein-like protein and a photosystem II D1 protein respectively, were found in 47 libraries.

### Unigene set annotation

#### BLASTN against cacao ESTs

The unigene dataset was used to detect how many cacao sequences had not been already described in public databases. To answer this question, we collected all 2539 *T. cacao *unique sequences already published by the Dana Farber Cancer Institute (DFCI) gene index [27] and we did a BLASTN search against our unigenes. An e-value cutoff of 1e^-50 ^was used to ensure that only highly similar sequences were detected. A total of 3901 unigenes produced a significant hit with 1788 unique sequences from the DFCI gene index, therefore these sequences may correspond to *T. cacao *sequences already published or may match different parts of the gene index sequences. They may be also produced by closely related genes (multigenic families). Finally, 44,693 unique sequences did not produce a significant hit, therefore these sequences may be new.

#### BLASTX and BLASTN annotation

**Figure 3:**
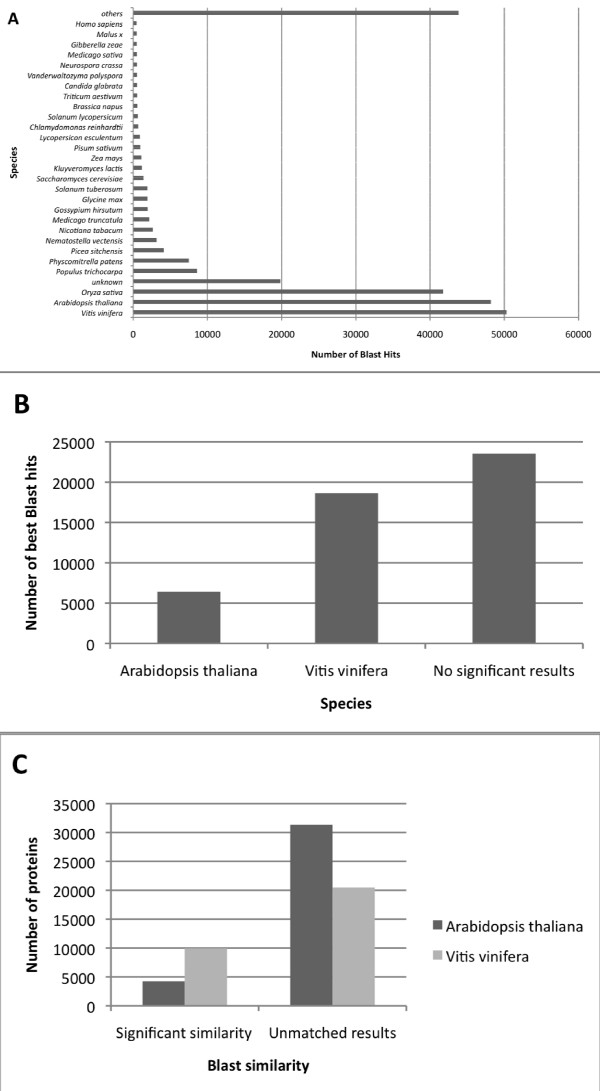
**Species distribution among the Blast results of *T. cacao *unigenes.** A – Distribution of species represented in the 10 first Blast hits against NCBI Non redundant protein database. B – Number of best Blast hits against *Arabidopsis thaliana *and *Vitis vinifera *proteomes. C – *Arabidopsis thaliana *(black columns) and *Vitis vinifera *(grey columns) proteome coverage.

The unigenes were first translated into amino-acid sequences and then searched for similar protein with the BLASTX program using an e-value cutoff of 1e-5 against the non-redundant protein sequence database (NR) with entries from GenPept, Swissprot, PIR, PDF, PDB and NCBI RefSeq. The 10 best hits were retained for the annotation, providing an annotation for 27,245 cacao sequences (56.1%). The 43.9% of the unigenes that did not have any match were searched for similar nucleotide sequences from the Genbank nucleotide collection NT with the BLASTN program. An e-value cutoff of 1e-5 was also used and the 10 best hits were used for the annotation. 2604 unigenes exhibited a significant similarity with nucleotide sequences providing a BLASTX or BLASTN annotation for 29,849 unigenes. The 10 BLASTX hits were used to classify the unigenes according to the species associated with the annotation (Figure 3A). A total of 140,270 hits (56%) involved proteins from *Vitis vinifera*, *Arabidopsis thaliana *or *Oryza sativa*, while 1955 hits involved proteins from *Gossypium hirsutum*, a closely related species from the Malvaceae family [28]. Although fewer protein sequences from *Vitis vinifera *than from *Arabidopsis thaliana *(54,395 and 58,061 respectively) were present in the non redundant database we used for BLASTX, and although the evolutionary distance between *Vitis vinifera *and *Theobroma cacao *is higher than the distance between *Arabidopsis thaliana *and *Theobroma cacao *[28], we found more similarities with *Vitis vinifera *(50,315 hits) than with *Arabidopsis thaliana *(41,766 hits).

To further investigate this unexpected result we compared with the BLASTX program the cacao unigenes dataset against the two proteomes of *Arabidopsis thaliana *and *Vitis vinifera *(Figures 3B, C). For each Blast result, we selected the species found in the first hit having an expected value lower than 1^e-15 ^to detect similar sequence. A total of 25,049 *Theobroma cacao *sequences (56%) presented at least a significant hit with an *Arabidopsis thaliana *or *Vitis vinifera *protein. The results showed that 18,643 *Theobroma cacao *sequences presented a higher similarity to the *Vitis vinifera *proteome whereas only 6406 *Theobroma cacao *sequences presented a first Blast hit similar to the *Arabidopsis thaliana *proteome (Figure 3B). Moreover, it was determined that these first significant hits involved 9943 *Vitis vinifera *proteins (33% of the proteome) and 4246 *Arabidopsis thaliana *proteins (12% of the proteome) (Figure 3C).

These surprising results suggest that the genes expressed in *Theobroma cacao *are more similar to *Vitis vinifera *proteins than to those of *Arabidopsis thaliana*. These findings could be explained by the fact that *Theobroma cacao *and *Vitis vinifera *are both fruit trees. This idea could be supported by the large amount of Blast hits found with other tree crops such as *Populus trichocarpa *(8605 Blast hits), despite a small number of non redundant proteins in the databases for this species.

#### Gene Ontology annotation

**Figure 4:**
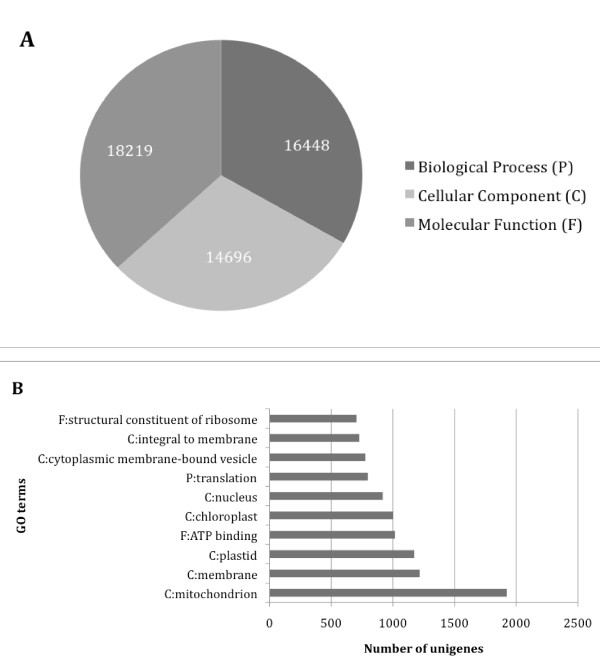
**Gene Ontology annotation results.** A – Distribution of the unigenes among the main Gene Ontology categories (Biological Process, Cellular Component and Molecular Function). B – Distribution of the unigenes among the 10 best Gene Ontology terms.

We used BLAST2GO [29], a program that retrieves GO terms based on BLAST definition, to assign gene ontology (GO) annotation [30] to the unigene dataset. To best exploit GO results, we built a local AmiGO browser [31]. A total number of 49,364 annotations were found and 16,364 unigenes were characterized by at least one annotation. These annotations were distributed among the main GO categories into 16,448 Biological Process (P), 14,696 Cellular Component (C) and 18,219 Molecular Function (F) (Figure 4A). The most abundant high-level direct GO counts within these categories were C: mitochondrion (1924), C: membrane (1218), C: plastid (1173), F: ATP binding (1017) and C: chloroplast (1001) (Figure 4B).

### Genes involved in defense and resistance mechanisms

Some of the libraries provide an important resource to study plant/pathogens interactions. Using the annotations provided by Blast and Gene Ontology, we specifically focussed on genes known to play a crucial role in plant pathogen resistance and defense mechanisms [32]. Using the AmiGO browser, we identified 1001 gene product associations to "response to stress" (GO:0006950). Both searches with Blast result and Gene Ontology annotation resulted in the identification of unigenes similar to known proteins involved in resistance or defense mechanisms such as LRR-NBS [33] (8 contigs and 32 singletons), chitinase [34] (19 contigs and 37 singletons), 1–3 beta glucanase [35] (5 contigs and 7 singletons) or pathogenesis-Related protein (24 contigs and 24 singletons).

Other genes related to resistance/defense mechanisms were also found more specifically in libraries produced from pathogen infected tissues, such as those involved in regulation of pathogen-induced genes like transcription factors (6 contigs and 7 singletons), in signal transduction (like MAPKinase with 5 contigs and 3 singletons) or in the cell death program.

The identification of a unigene set gathering sequences from all genes known to be involved in plant resistance and defense mechanisms, and the construction of a corresponding microarray could constitute a valuable tool to progress in the understanding of plant/pathogens interactions.

### Genes involved in particular metabolic pathways or biological activities

To check the representativeness of our EST collection, we looked for ESTs encoding proteins known to be involved in the flavonoid and the terpene pathways, already studied in other plant species, and at the basis of important traits of interest in *T. cacao*. Generally, polyphenols play a major role in chocolate quality, acting as colour precursors or taste agents [36]. Moreover, they are strongly implicated in health benefits associated with chocolate consumption [37-40].

#### The flavonoid pathway

The flavonoid pathway has been already studied in several plants [41]. In *T. cacao*, this pathway is the source of numerous essential components for human health benefits of chocolate [37-40] and resistance against pathogens [42].

**Figure 5:**
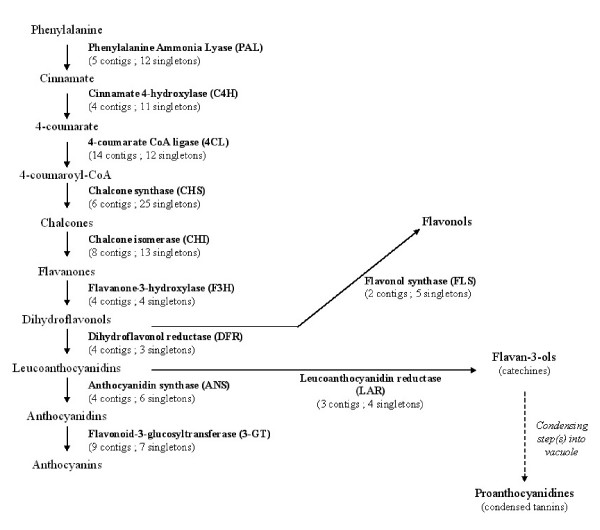
**Schematic overview of the general flavonoid biosynthesis pathway (according to Schijlen et al., 2004; Marles et al., 2003).** The number of contigs and singletons present in our EST dataset was added between brackets for each enzyme.

Gene Ontology analysis highlighted 99 EST sequences implicated in "phenylpropanoid biosynthetic process" (GO:0009699), most of them implicated in flavonoid biosynthesis. For example, the GO analysis, together with keyword ESTtik database searching (see material and methods) into Blast Results allowed us to find sequences encoding phenylalanine ammonia lyase (5 contigs and 12 singletons), cinnamate-4-hydroxylase (4 contigs and 11 singletons), the 4-coumarate-CoA ligase (14 contigs and 12 singletons), chalcone synthase (6 contigs and 25 singletons) and chalcone isomerase (8 contigs and 13 singletons), all major enzymes of the general flavonoid pathway (Figure 5). Most specific enzymes, implicated in anthocyanin biosynthesis (flavanone-3-hydroxylase, dihydroflavonol reductase, anthocyanidin synthase, flavonoid-3-glucosyltransferase) were also represented in this *T. cacao *EST resource.

#### The terpene pathway

**Figure 6:**
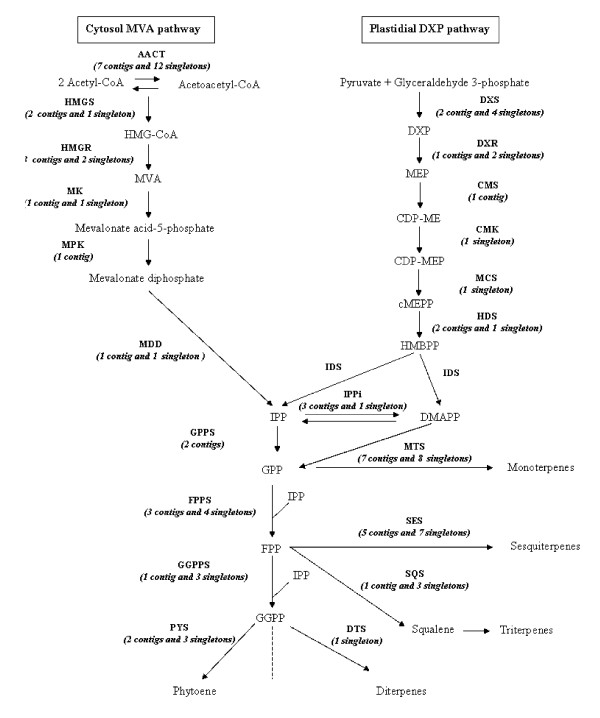
**The biosynthesis pathway of isoprenoïdes.** (according Liu *et al*., 2005). Pathway Mevalonate (MVA) cytoplasmic in left and pathway 1-deoxyxylulose-5-phosphate (DXP) chloroplastic in right. **AACT**, acetoacetyl-coenzyme A (CoA) thiolase; **CMS**, 2-*C*-methyl-*D*-erythritol 4-phosphaate cytidyl transferase; **DTS**, diterpene synthase; **DXR**, 1- deoxy-*D*-xylulose 5-phosphate reductoisomerase; **DXS**, 1-deoxy-*D*-xylulose 5-phosphate synthase; **FPPS**, farnesyl diphosphate synthase; **GGPPS**, geranylgeranyl diphosphate synthase; **GPPS**, geranyl diphosphate synthase; **HMGR**, 3-hydroxy-3-methylglutaryl coenzyme A (HMG-CoA) reductase; **IPPi**, isopentenyl diphosphate isomerase; **MTS**, monoterpene synthase; **SES**, sesquiterpene synthase; **SQS **squalene synthase; **MK**, mevalonate kinase; **MPK**, mevalonate-5-phosphate kinase; **CMK**, 4-(cytidine 5'-diphospho)-2-*C*-methyl-*D*-erythritol kinase; **MDD**, mevalonate diphosphate decarboxylase; **IDS**, isopentenyl diphosphate/dimethylallyl diphosphate synthase; **MCS**, 2-*C*-methyl-*D*-erythritol 2,4-cyclodiphosphate synthase; **HDS**, 1-hydroxy-2-methyl-2-(*E*)-butenyl 4-diphosphate synthase; **PSY**, phytoene synthase; **HMGS**, HMG-CoA synthase; **HMG-CoA**, 3S-hydroxy-3-methylglutaryl coenzyme A; **DXP**, 1-deoxy-*D*-xylulose 5-phosphate; **MVA**, 3R-Mevalonic acid; **MEP**, 2-*C*-methyl-*D*-erythritol 4-phosphate; **CDP-ME**, 4-(cytidine 5'-diphospho)-2*C*-methyl-*D*-erythritol; **CDP-MEP**, 4-(cytidine 5'-diphospho)-2*C*-methyl-*D*-erythritol 2-phosphate; **cMEPP**, 2*C*-methyl-*D*-erythritol 2,4-cyclodiphosphate; **DMAPP**, Dimethylallyl diphosphate; **HMBPP**, 1-hydroxy-2-methyl-2-(*E*)-butenyl 4-diphosphate; **IPP**, isopentenyl diphosphate; **GPP**, geranyl diphosphate; **FPP**, farnesyl diphosphate; **GGPPS**, geranylgeranyl diphosphate. The number of contigs and singletons present in our EST dataset was added between brackets for each enzyme.

Terpenoid compounds, synthesized in the isoprenoid pathway (Figure 6), are compounds of importance for specific scent and aromatic qualities of chocolates classified as "fine and flavor". For example, linalool, a monoterpenol, is found in high quantity in Arriba Nacional varieties from Ecuador and in some Criollo clones from Venezuela [11,43,44]. Linalool, together with other volatiles, could be responsible for the typical floral aroma [45] of these chocolates.

One of our goals was to identify enzymes involved in the terpenoid pathway that could be responsible for linalool content variations among Nacional clones. As a first step we identified sequences encoding isoprenoid pathway enzymes (42 contigs and 55 singletons). The final step enzyme for linalool synthesis, linalool synthase, was represented by 2 contigs and 4 singletons. Nearly all enzymes reported to be involved in this biochemical pathway were present in our ESTtik database, allowing the analysis of the *T. cacao *terpene pathway based on oligonucleotide microarrays derived from these ESTs.

The fact that nearly all of the genes involved in these two pathways as described in other plant species were identified in ESTs from our collection demonstrates the high level of representation of this resource and suggests that the majority of cacao genes have been sampled. Thus, this EST collection offers a comprehensive resource to search for candidate genes involved in quality traits and other important agronomical traits variation.

#### Production of SSR and SNP markers

Molecular markers derived from ESTs are part of, or adjacent to genes, and therefore they provide an efficient means of gene mapping.

Simple Sequence Repeats (SSRs) were identified in the unigene dataset with the MISA pipeline [46]. In this study, SSRs were defined as dimers with at least 6 repetitions and trimers, tetramers, pentamers and hexamers with at least 5 repeats. Microsatellites were considered compound when two SSRs were not separated by more than 100 bp. A total of 2252 SSRs were identified as 2164 unigenes, and 204 unigenes had more than 1 SSR. Dimers and trimers were the most common types (Table 2) and represented 94.2% of SSRs found in unigenes. The distribution of all possible dimer and trimer motifs found in the unigenes is listed in Table 3. The poly(AG)n and poly(AAG)n groups were the most abundant motifs in *T. cacao *unigenes.

**Table 2:** Distribution of motifs length in SSRs dataset

**Motif Length**	**Number of SSRs**	**Frequency**
2	1132	50.3
3	857	38.1
4	82	3.6
5	35	1.6
6	14	0.6
compound	132	5.9

**Table 3:** Distribution of dimers and trimers motifs in SSRs dataset

**Group**	**Motif**	**Number of SSRs**	**Frenquency**
AC	AC/CA/GT/TG	55	2.4
AG	AG/CT/GA/TC	754	33.5
AT	AT/TA	323	14.3
CG	CG/GC	0	
			
AAT	AAT/ATA/TAA/ATT/TTA/TAT	121	5.4
AAG	AAG/AGA/GAA/CTT/TTC/TCT	308	13.7
AAC	AAC/ACA/CAA/GTT/TTG/TGT	54	2.4
ATG	ATG/TGA/GAT/CAT/ATC/TCA	117	5.2
AGT	AGT/GTA/TAG/ACT/CTA/TAC	9	0.4
AGG	AGG/GGA/GAG/CCT/CTC/TCC	81	3.6
AGC	AGC/GCA/CAG/GCT/CTG/TGC	79	3.5
ACG	ACG/CGA/GAC/CGT/GTC/TCG	9	0.4
ACC	ACC/CCA/CAC/GGT/GTG/TGG	66	2.9
GGC	GGC/GCG/CGG/GCC/CCG/CGC	13	0.6

For each SSR identified, if possible, 3 couples of primers were defined using Primer3 [47]. A total of 5265 flanking sequences were designed and it was possible to define at least one couple of primers for 1755 SSRs.

The exploration of redundant ESTs in contigs was shown to be a valuable resource of Single Nucleotide Polymorphisms (SNP) [48]. SNPs were detected using QualitySNP [49] pipeline from unigene contigs. We assumed that contigs with at least 100 members contained paralogous sequences [50,51] therefore we selected 4818 contigs that contained at least 4 sequences but no more than 100 sequences. A preliminary study assembled 5246 SNPs into 2012 contigs. Transitions (A/T-G/C) represented 54.2% of the SNPs found, transversions 32.1% and InDels 13.7%.

## Conclusion

The present assembly of 149,650 *T. cacao *ESTs produced from 56 cDNA libraries constructed from different organs and environmental conditions is the largest transcriptome dataset produced so far for *T. cacao*, and among the largest ones generated for any tree fruit crop. It provides a major resource for *cacao *genetic and functional genomic analyses of important *T. cacao *traits, with the identification and annotation of 48,594 different putative transcripts.

The improved knowledge of the *T. cacao *transcriptome will enhance our understanding of main disease resistance mechanisms and will be useful to improve new varieties and establish a sustainable *T. cacao *resistance to pests and diseases. Towards this goal, a large number of cDNA libraries have been produced from *T. cacao*/pathogens or pest interactions, and an important set of unique transcripts homologous to genes known in other species involved in defense and resistance mechanisms have been identified in the whole EST collection using keywords and Gene Ontology tools. It provides a cDNA resource available for the broad scientific community and suitable for cDNA-based microarray analyses.

This collection of ESTs also provides a valuable framework for the discovery of candidate genes involved in chocolate quality traits. Tested for two distinct metabolic pathways, this collection displays a good representation of the *T. cacao *transcriptome involved in quality trait elaboration and will allow the comparative analysis of contrasting genotypes for *T. cacao *qualities to better understand the genetic basis of quality.

This EST collection also will provide a large number of genetic tools, such as SSR and SNP markers, which will be used to construct high density gene maps, facilitating the integration of genetic and genomic approaches to discover the genes that effect trait variations, and also facilitating the sequence assembly in further activities of whole *T. cacao *genome sequencing.

Finally, the assembly and annotation associated will also provide a valuable resource for future investigation of *T. cacao *evolutionary genomics with related species such as *Gossypium hirsutum *or *Arabidopsis thaliana*.

## Methods

### Material used for libraries construction

In total, 56 different libraries were constructed. The organs and *T. cacao *genotypes used for cDNA construction, and the treatments carried out on these organs are reported in Table 1.

Most of the libraries were constructed from 2 genotypes:

- Scavina 6 (SCA6) is a self incompatible Forastero genotype originating from the Upper Amazonian region of Peru. SCA6 is highly resistant to *Phytophthora *species and *Moniliophthora perniciosa *diseases. It has been widely used in the breeding programs.

- ICS1 is a self compatible Trinitario genotype, a hybrid involving Criollo, the first *T. cacao *variety domesticated in Central America, and a Forastero variety originated from the Lower Amazonia of Brazil; ICS1 is known for its large beans and good quality traits. This clone was used for RNA production during the different stages of development of the *T. cacao *seeds.

A post harvest treatment is generally applied to *T. cacao *seeds to develop chocolate, involving fermentation steps, drying and torrefaction. Tissues from ICS1 Seeds were collected during the first 2 days of fermentation to construct cDNA libraries.

Other genotypes were used more specifically to represent particular traits or genetic origins:

- Jaca is a Brazilian Forastero genotype from the Upper Amazonian region, and resistant to *Ceratocystis fimbriata*. Inoculation was done according to Silva *et al*. [52]

- B97 C-C-2 is a pure and homozygous Criollo genotype. This material was collected in Belize [53] by a mission conducted by the CRU (Cocoa Research Unit, Univ. West Indies, Trinidad) in conjunction with The Maya Mountain Archaeological Project (MMAP – Cleveland State Univ.) and is now grown in the international collection of CRU.

- GU255V is a genotype originated from French Guyana, resistant to *Phytophthora palmivora*. Inoculation was done according to Tahi *et al*. [54]

- PNG seedlings are from a progeny produced in Papua New Guinea from the cross of two hybrids: 17/3-1 × 36/3-1, and segregating for *Phytophthora resistance*. Inoculation was done according to Tahi *et al*. [54]

- UF676 is a Trinitario genotype tolerant to mirids. Insect attack was done using protocol described by Babin *et al*. [55].

- P7, IMC47, UPA134 are Forastero genotypes originated from the Upper Amazonian region of Peru, known for their resistance to *Phytophthora palmivora or P. megakarya*. Inoculation was done according to Tahi *et al*. [54]

- UF 273 is a Trinitario genotype resistant to *Moniliophthora *rorer. Inoculation was done according to Khun *et al*. [56]

- 33–49 and BE240 are Nacional genotypes from Ecuador known for their aromatic and floral taste.

SSH libraries or direct libraries were constructed from these genotypes. More information related to these genotypes is available through the International Cocoa Germplasm Database [57].

Drought Stress Libraries were constructed from total RNA isolated from leaves and roots of Scavina 6 plants that were initially grown under standard conditions in a greenhouse [58]. Rooted cuttings were generated and grown to about 6 months old, then were moved into a Conviron growth chamber and were not watered until leaves were visibly wilted (approx 36 hours) at which time tissues were flash frozen in liquid nitrogen.

### RNA Extraction

Plant tissues were frozen in liquid nitrogen or placed in RNA stabilization reagent (RNA *later*™, Qiagen) and stored at -20°C before RNA extraction. Approximately 100 mg of plant tissues were crushed in liquid nitrogen with poly-vinyl-poly pyrrolidone. The powder was transferred in a tube containing 1 ml of extraction buffer " TE3D " (14.8 g EDTA, 84.4 g Tris, 20 g Nonidet P-40, 30 g lithium dodecyl sulfate, 20 g sodium deoxycholate, 95 ml H2O) [59]. After 15 min incubation at room temperature, 1 ml of sodium acetate (3 M) and one volume of chloroformisoamyl alcohol (24:1) were added. Purification of the aqueous phase was carried out following centrifugation by adding one volume of mixed alkyl tri-ethyl ammonium bromine solution (2% MATAB, 3 M NaCl) followed by 15 min at 74°C. The residual polysaccharides were then eliminated by addition of one volume of chloroformisoamyl alcohol (24:1) and centrifugation; the aqueous phase was precipitated by the addition of one volume of isopropyl alcohol. After centrifugation, the pellet was resuspended in 50 μl of ribonuclease free water containing 1 μl of ribonuclease inhibitor (RiboLock™, Fermentas).

RNA samples from cacao tissues were isolated following the procedure of Charbit *et al *[59] with modifications. Following DNase treatment (DNase I, Fermentas), RNA was then extracted with the phenolchloroformisoamyl alcohol (25:24:1) step and precipitated with one-tenth volume of 3 M sodium acetate, pH 5.3, and 2.5 volumes of 100% ethyl alcohol. An aliquot of RNA was then run by elecrophoresis on a 1.2% agarose gel and stained with ethidium bromide to confirm RNA integrity.

### Construction of full-length enriched cDNA library

First strand cDNA were synthesized using the Clontech BD SMART PCR cDNA Synthesis KIT (cat No 634902) as recommended by the supplier. 0.5–1 μg of total RNA was incubated at 72°C for 2 min with 1 μl 3' BD SMART CDS Primer II A (12 μM) and 1 μl BD SMART II A Oligonucleotide (12 μM) in a total volume of 5 μl. Then 2 μl 5× First-Strand Buffer, 1 μl DTT (20 mM); 1 μl dNTP Mix (10 mM of each dNTP), 1 μl BD PowerScript Reverse Transcriptase were added and the mix was incubated at 42°C for 1 hour in an air incubator. According to Glen K Fu (2003) [60], 3 μl Biotin-dATP (Invitrogen), 3 μl Biotin-dCTP (Invitrogen), 1 μl 5'-NVVVVV-3' primer 30 μM (50 ng), 2 μl 5× First-Strand Buffer, 1 μl BD PowerScript Reverse Transcriptase were added, and the mix was kept at 42°C for 30 min. For capture of the unfinished strand, the reaction was mixed with 600 μl of Streptavidine MagneSphere Paramagnetic Particles (Promega) and eluted as recommended by the supplier.

A 2 μl aliquot from the first strand synthesis was used for the cDNA Amplification by LD PCR (Clontech). Each reaction was performed with 80 μl deionized water, 10 μl 10× BD Advantage 2 PCR Buffer, 2 μl 50× dNTP Mix (10 mM of each dNTP), 4 μl 5' PCR Primer II A (12 μM), 2 μl 50× BD Advantage 2 Polymerase Mix in a 98 μl total volume. The PCR reaction consisted of 18 to 25 PCR cycles at 95°C for 15 sec, 65°C for 30 sec, 68°C for 6 min, following with a final extension at 70°c for 10 min.

After comparison of fragment sizes with those of model species (rice and *Arabidopsis*), fragment sizes of some cDNA libraries were improved using cDNA size fractionation. These libraries were submitted to an "agarase step" [61] after 18 cycles PCR. Double-stranded cDNA was separated on 1% low-melting agarose gel and the DNA ladder "lane" was stained and photographed with a ruler. Two size fractions (< 1.2 kb and > 1.2 kb) were excised from the unstained cDNA "lane" based on the DNA ladder "lane". cDNAs were extracted from the gel slices with agarase (Fermentas) according to the supplier instructions. After a gelase digestion, the cDNA was precipitated with one volume of isopropanol. The pellets were dried and suspended in ribonuclease free water. Four to five additional PCR cycles were performed in order to improve the efficiency of ligation in pGEM^®^-T Easy Vector.

For SSH cDNA libraries: The procedure was performed with the PCR-Select cDNA Substraction kit (Clontech) according to the manufacturer's recommendations with slight modifications. The cDNA generated from the SMART procedure was restricted with 15 U of *Rsa*I (Fermentas) and the two aliquots of the tester cDNA were ligated to adaptors 1 and 2R, respectively, with 30 U of T4 DNA ligase (Fermentas). The PCR mixture enriched for differentially expressed sequences was cloned using pGEMT (Promega) as mentioned above.

One μl of the second strand product was cloned in pGEM^®^-T Easy Vector Systems (Promega) and transformed by electroporation in the DH10B T1 resistant strain of *Escherichia coli *(Invitrogen); transformation products were plated on LB-ampicillin agar plates and incubated overnight at 37°C. White colonies were picked using a Qpix 2 XT biorobot (Genetix) and stored in 384 well plates at -80°C.

### Sequencing

All clones were end-sequenced using either Forward or Reverse M13 primers. The sequencing reactions were performed with Applied Biosystems BigDye V3.1 kits, and were resolved on ABI3730xl DNA Analysers

### Sequence processing

**Figure 7:**
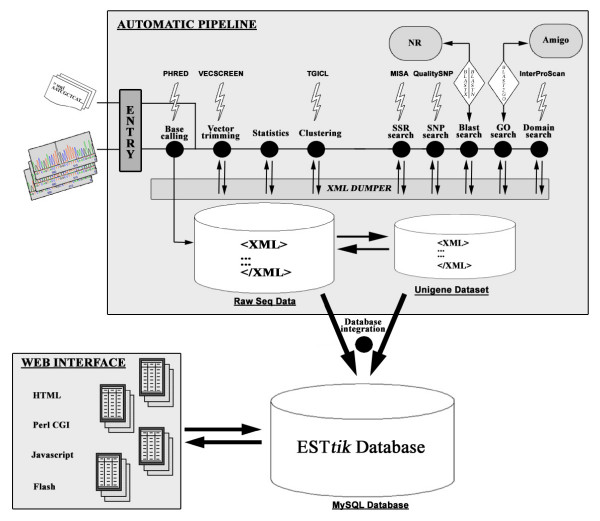
Schematic overview of the ESTtik information System.

Sequences were managed and stored using our own tool called Expressed Sequence Tag Treatment and Investigation Kit (ESTtik) which is an information system that contains a pipeline for processing, a database and a web site for querying data (Figure 7). The ESTtik pipeline program is a set of Perl packages which contain a main program related to 9 modules in charge of completing different processings. The pipeline executes a series of programs to assess quality and nucleotides from chromatograms, then edits, and assembles the input DNA sequence information into a non-redundant data set. This unigene is then searched for microsatellites and SNPs. It is used as input for an annotation against public databases including an extraction of Gene Ontology terms [30]. All the results produced by automatic processing are finally stored into XML files. The information collected from individual program modules of the pipeline is stored into a MySQL database. The database model was specially designed using the UML technology to fit data. To visualize Blast [62] results, annotations and to search for sequences by gene keywords or GO terms, the ESTtik database records can be accessed using 7 query pages combining PerlCGI, HTML, Javascript and Flash technologies.

The software Phred [63] was used for base calling linked to Vecscreen [64] for vector and adapters trimming. Cleaning of sequences was performed with the standalone low complexity filter mdust and bioperl modules. Each forward and reverse ESTs were individually assembled with the CAP3 program, using an overlap percent identity cutoff of 65 (p) and an overlap length cutoff of 20 (o).

Special attention has been paid to the global assembly of ESTs, in order to obtain the most representative transcription units. The TGI Clustering tools (TGICL) were used because they provide an optimized protocol for the analysis of EST sequences [65]. This package performs a clustering phase (using megablast) without multiple alignments, and then creates contigs (consensus sequences) with the assembly program CAP3. Many parameters were tested and because we had clusters made of ESTs coming from several highly expressed genes, we increased the clustering and assembly stringency. For the clustering step, we used a minimum percent identity for overlaps (p) of 94, a minimum overlap length (l) of 30, a maximum length of unmatched overhangs (v) of 30. For the assembly, we used a specify overlap percent identity cutoff (p) of 93.

### Annotation

Similarity searches were performed with the standalone version 2.2.16 of BLAST [62] against non redundant proteins and nucleotides. The XML Blast output was used and parsing of results was performed with the Bio::SearchIO module of Bioperl toolkit [66].

We built a local Blast2GO MySQL database and we first used the Blast2GO program [29] with default parameters to assign Gene Ontology (GO) terms to the unigenes based on the BLAST definitions. To best exploit GO annotations, results were integrated into a local AmiGO browser and database.

### Molecular markers

SSRs searches were performed with MIcroSAtellite identification tool (MISA) [46] and primers designed with Primer3 software [47].

The QualitySNP pipeline [49] was used for detecting single nucleotide polymorphisms in the unigenes.

### Data availability

Sequence data, molecular markers and high quality annotation will be integrated into CocoaGen DB [67], a Web portal developed for combining *T. cacao *molecular genetic and genomic information from TropgeneDB [68] and phenotypic data from The International Cocoa Germplasm Database [57]. The individual ESTs of the 56 libraries were deposited in the EMBL database under accession CU469588 to CU633156.

## Authors' contributions

XA carried out bioinformatic tasks and drafted the manuscript. OF participated in construction of cDNA libraries. PW carried out EST sequencing. KG contributed to library construction and vegetal material preparation/inoculation. TL contributed to library construction. XS contributed to library construction and replication and sequence analyses. AMR contributed to library construction. CDS contributed to bioinformatic analyses. JC contributed to library construction and vegetal material preparation/inoculation. MA contributed to sequence analyses. DK contributed to library construction and vegetal material preparation/inoculation. JV contributed to library construction and vegetal material preparation/inoculation. BC contributed to bioinformatic analyses. GL contributed to vegetal material preparation/fermentation. RB contributed to vegetal material preparation/inoculation. OS contributed to vegetal material preparation/inoculation. MD contributed to vegetal material preparation/inoculation. MG contributed to library construction. MR contributed to bioinformatic analyses. LA contributed to vitroculture production. RM contributed to vitroculture production. WP contributed to vegetal material preparation/inoculation. RS contributed to library construction. MG participated in general discussion and management. ER participated in general discussion and management. SM contributed to library construction. CL coordinated the Project, drafted the manuscript and participated in library construction.
